# Gut and oral microbiome profiles in patients with obesity and ischemic heart disease

**DOI:** 10.3389/fcimb.2025.1695279

**Published:** 2025-12-12

**Authors:** Antonina Starodubova, Georgy Leonov, Nataliya Shaposhnikova, Yurgita Varaeva, Elena Livantsova, Denis Fotin, Tatiana Kirichenko, Mariam Bagheri Ekta, Yuliya Markina, Stanislav Koshechkin, Alexander Orekhov, Dmitry Nikityuk

**Affiliations:** 1 Department of Cardiovascular Pathology and Diet Therapy, Federal Research Center of Nutrition, Biotechnology and Food Safety, Moscow, Russia; 2 Institute of Clinical Medicine, Pirogov Russian National Research Medical University, Moscow, Russia; 3 Petrovsky National Research Center of Surgery, Moscow, Russia; 4 Nobias Technologies, Moscow, Russia; 5 Institute of General Pathology and Pathophysiology, Moscow, Russia

**Keywords:** gut microbiome, oral microbiome, 16S rRNA sequencing, obesity, IHD

## Abstract

**Background:**

Ischemic heart disease (IHD) and obesity are major contributors to global mortality. Both conditions are linked to systemic inflammation, dyslipidemia, and microbiota alterations. This study examines the relationship between the composition of the gut and oral microbiota, obesity, and IHD to gain insight into the interconnections between these factors.

**Methods:**

The study included 182 participants divided into four groups based on obesity and IHD status. Anthropometric and biochemical analyses were performed. Oral and gut microbiomes were analyzed using 16S rRNA sequencing.

**Results:**

Obesity and IHD were associated with distinct microbiota compositions. Obesity-IHD subjects showed elevated levels of gut *Streptococcus, Intestinibacter*, alongside reduced *Citrobacter, Ruthenibacterium, Parabacteroides, and Flavonifractor*. The oral microbiota exhibited decreased *Alloprevotella, Capnocytophaga, Prevotellamassilia, and Campylobacter* in Obesity-IHD. Correlation analysis highlighted associations between microbial taxa (e.g., *Blautia*, *Oscillibacter*) and clinical parameters like BMI, blood pressure, and cholesterol.

**Conclusions:**

This study demonstrates that obesity and IHD are linked to unique microbiota alterations. Microbial dysbiosis may contribute to the pathogenesis of these conditions and should be considered as a therapeutic target in the development of personalized treatment strategies of the obesity and associated cardiovascular complications.

## 1 Introduction

Ischemic heart disease (IHD, also known as coronary artery disease) and other cardiovascular diseases, including stroke, are responsible for approximately 17.9 million deaths annually, representing 32% of global mortality (Luo et al., 2024b). IHD is characterized by narrowing or blockage of the coronary arteries, primarily due to atherosclerosis (Severino et al., 2020). Major modifiable risk factors for IHD include high systolic blood pressure, dietary risks, dyslipidemia (high LDL cholesterol), obesity (high body mass index), high fasting plasma glucose, tobacco, air pollution, lack of physical activity, excessive alcohol consumption, and others (Ng et al., 2020; Roth et al., 2020).

Furthermore, conditions such as metabolic syndrome and its individual components, including obesity, diabetes, and dyslipidemia, have been demonstrated to markedly elevate the risk of developing IHD (Montazerifar et al., 2016; Niwa, 2021). Obesity is well-established as a major risk factor for IHD, primarily due to its association with dyslipidemia, hypertension, and insulin resistance (Thomsen and Nordestgaard, 2014). A number of studies have demonstrated that both overweight and obesity, in the absence of metabolic syndrome, are associated with an elevated risk of myocardial infarction and ischemic heart disease in the general population (Pham et al., 2024; Sedaghat et al., 2024).

The wide range of current massive sequencing techniques has enabled the identification of the profile of the intestinal microbiota and the elucidation of its impact on human metabolism. This has led to the recognition of the microbiota's fundamental role in health and diseases (Kobiyama and Ley, 2018). The relationship between microbiota and body weight control has been the subject of investigation, however no definitive causality or association between these two factors has been established (Boscaini et al., 2021). A number of studies have demonstrated structural differences in the intestinal microbiota between individuals with and without obesity (Companys et al., 2021; Duan et al., 2021; Pinart et al., 2022). Some studies have suggested that the microbiome of obese people contains a higher proportion of the *Firmicutes* (*Bacillota*) phylum and a lower proportion of *Bacteroides* (*Bacteroidota*) (F/B ratio) (Kasai et al., 2015; García-Gamboa et al., 2024). Nevertheless, there are studies that do not support this assertion (Pinart et al., 2021a). Several studies have demonstrated the contribution of specific microbial species to the development of obesity. It is therefore postulated that a reduction in the abundance of *Akkermansia muciniphila* is a characteristic feature of obese individuals (Depommier et al., 2019; Abuqwider et al., 2021). The composition of the microbiota is influenced by a variety of factors, including nutrition, physical activity, and medication intake (Mirzahosseini et al., 2024).

Numerous studies have demonstrated significant differences in the composition of the oral microbiome between normal-weight and obese populations (Wu et al., 2018; Yang et al., 2019). Some of these studies identified similar obesity-associated oral and gut microbial rearrangements (de Andrade et al., 2020). Specifically, some research has shown an increased abundance of *Firmicutes*, a higher *Firmicutes* / *Bacteroides* ratio, and reduced microbial diversity in the oral microbiome of obese individuals (Sohail et al., 2019; Mohamed Qadir and Assafi, 2021). Recent findings indicate that gut microbiota-derived factors, such as short-chain fatty acids (SCFAs) and trimethylamine oxidase (TMAO), play a pivotal role in regulating systemic inflammation, intestinal permeability, and immune activation. These factors contribute to the pathogenesis of cardiovascular diseases, including myocardial infarction, hypertension, atherosclerosis, and atrial fibrillation (Leonov et al., 2025a). A recent study has found significant differences in the abundance of several genera in both oral and intestinal microbiome patterns in obese participants (40–59 years) compared to lean participants (Stefura et al., 2021).

Dental medicine research has revealed that the oral microbiome of patients with obesity is characterized by an increase in traditional periodontal pathogens, reflecting a well-established association between periodontitis and obesity (Leonov et al., 2025b). Oral microbiome dysbiosis has also been linked to cardiometabolic health, with proposed mechanisms including effects on chronic inflammation and blood pressure (Tonelli et al., 2023).

The aim of this study was to examine the correlation between the structure of the intestinal and oral microbiome and the prevalence of conditions such as obesity and established coronary heart disease, as well as their co-occurrence.

## 2 Materials and methods

### 2.1 Subjects and study design

A total of 182 Caucasian subjects (42 men (23.1%), mean age 61.2 ± 6.5 years, mean BMI 35.6 ± 7.6 kg/m^2^) were included in this study. Participants were categorized into four groups based on obesity and the presence of ischemic heart disease (IHD): Control group: non-obese individuals without IHD (n=27); IHD group: non-obese individuals with IHD (n=22); Obesity group: obese individuals without IHD (n=59); and Obesity-IHD: obese individuals with IHD (n=74). All 96 IHD patients had a confirmed diagnosis based on coronary angiography data, of whom 55 had suffered acute coronary syndrome (ACS) or stroke.

**Figure 1:**
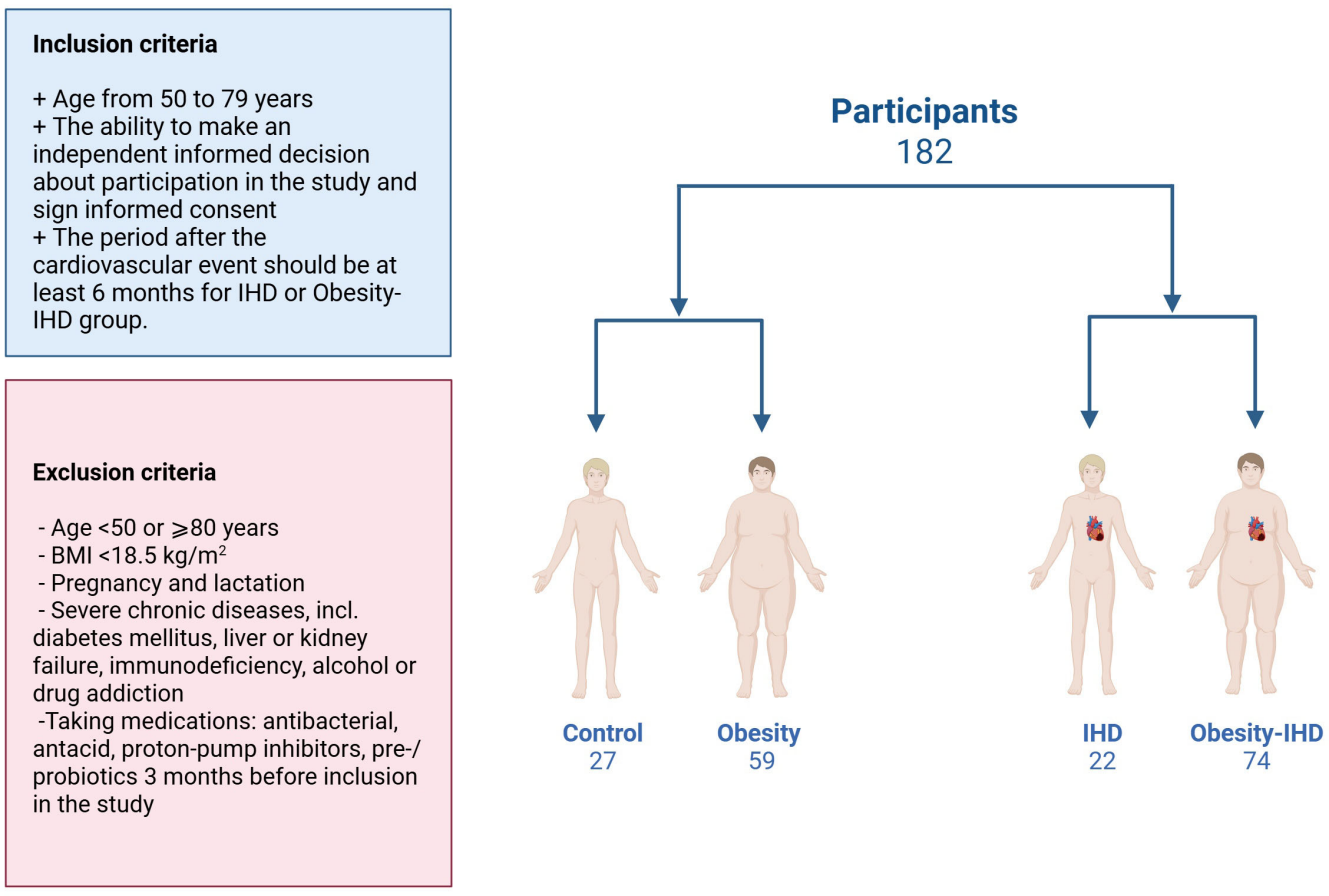
Flowchart visualizing participant recruitment. A total of 182 participants were enrolled in the study. Individuals were stratified according to the presence of obesity and IHD.

Ethical Approval was received in the Local Ethics Committee (protocol code N1/2021 dated on 08/FEB/2021). Participants were recruited in the period from March 2021 to March 2024. Informed consent was obtained from all subjects involved in the study. All participants underwent examination at the Nutrition Clinic of the Federal Research Centre of Nutrition, Biotechnology and Food Safety. The collected samples were analyzed in a de-identified manner to ensure the confidentiality of the participants. Inclusion and exclusion criteria were presented in **Figure 1**.

### 2.2 Anthropometry, body composition and biochemical indicators

Body weight and height were measured using a medical scale and stadiometer, respectively, and recorded in kilograms and meters. The body mass index (BMI) was calculated in accordance with standard procedures. Body composition parameters, including body fat mass (in kilograms), muscle mass (in kilograms), and fat proportion (in percentage), were assessed through bioimpedance analysis using the InBody 770 analyzer (Inbody Co., Ltd., Republic of Korea). Serum levels of total cholesterol (TC), low-density lipoproteins (LDL), high-density lipoproteins (HDL), triglycerides (TG) and glucose were measured in accordance with standard laboratory procedures on the KONELAB Prime 60i analyzer (Thermo Fisher Scientific, Waltham, MA, USA).

### 2.3 Fecal sample collection

Prior to sample collection, patients were instructed to refrain from the use of sorbents or laxatives, including magnesia and castor oil. Fecal samples were obtained *via* sterile collection tubes, frozen at a temperature of −40 °C.

### 2.4 Oral samples collection

Oral samples were collected in the morning, at least 8 hours after the participants’ last tooth brushing and food or liquid intake. Participants rinsed their mouths with sterile water and waited for approximately 5 minutes before providing saliva samples, which were collected using the spitting method (3–5 mL over 3 minutes) into sterile polypropylene tubes. Saliva samples were then pooled and stored at -80 °C until nucleic acid extraction.

### 2.5 DNA extraction, gut and oral microbiota composition profiling

The DNA was extracted from the oral microbiome samples using DNA extraction kits with an inhibitor removal stage (Nobias Technologies, Russia). The DNA was extracted from fecal samples using DNA extraction kits with a sample homogenization stage using solid-state microparticles and inhibitor removal (Nobias Technologies, Russia). The number of 16S gene replicates in the isolated DNA solution was estimated using quality control kits for the pre-analytical phase of metagenomic studies (Nobias Technologies, Russia). For amplification of the full-length 16S rRNA gene, 27F and 1492R primers (AGAGTTTGATYMTGGCTCAG and GGTTACCTTGTTAYGACTT, respectively), and a CFX 96 amplifier (Bio-Rad, USA) were used. The obtained PCR products were purified using Agencourt AMPure XP magnetic particles (Beckman Coulter Inc., USA). The quality of the obtained amplicons was assessed by electrophoresis in 1.5% agarose gel. Further preparation of amplicon libraries and sequencing was performed using NEB reagents: single-strand break and end repair “NEBNext FFPE Repair Mix” (M6630), “NEBNext End repair / dA-tailing Module” (E7546), and adapter ligation “NEBNext Quick Ligation Module” (E6056). All enzyme-dependent (intermediate) stages of library preparation were accompanied by the necessary sample purification using Agencourt AMPure XP magnetic particles (Beckman Coulter Inc., USA). The concentration of the resulting 16S rRNA libraries in solution was measured using a Qubit fluorimeter (Invitrogen, USA) using the dsDNA Quant-iTTM high-sensitivity assay kit (Thermo Fisher Scientific, USA). Purified libraries were mixed equimolarly according to the estimated concentrations. Sequencing was performed using kits manufactured by Oxford Nanopore Technologies: Ligation Sequencing Kit (SQK-LSK109), Flow Cell Priming Kit (EXP-FLP002) and Native Barcoding Expansion 96 (EXP-NBD196) PCR-free multiplexing kit. Sequencing was performed on a MinION instrument using an R9 series cell (FLO-MIN106). Guppy software (version 5.1.13) was used for basecalling, High accuracy basecalling mode was used as a model, and minimum quality cutoff was fixed at qscore=7. To assess possible contamination during sample preparation and to make appropriate corrections, a positive control sample consisting of 100% *Lactobacillus rhamnosus* was added to each batch of samples along with skin microbiota samples. Read quality was assessed using NanoFilt. Reads shorter than 1400 bp and with a quality score of less than 10 were excluded. Further analysis included samples for which at this stage there were at least 10000 reads. Representation tables were obtained by summing the representation of species belonging to the corresponding taxonomic group at the species, genus, family, and other levels.

### 2.6 Statistical analysis

The normal distribution of the data was assessed using the Kolmogorov-Smirnov test with Lilliefors correction. A chi-square test was used to calculate the frequency distributions, and a non-parametric Kruskal–Wallis test was used to calculate the differences in continuous variables between conception outcomes. R programming language, utilizing packages such as Phyloseq (data processing and statistics) and ggplot2 (visualization), was used to calculate alpha and beta diversity parameters and subsequent visualization. Statistical significance for alpha diversity was calculated using the Wilcoxon test (built-in R function), the Vegan package was used for beta diversity. Statistics for genera and species were calculated using the DESeq2 package, with P-values adjusted using the Benjamini-Hochberg procedure (padj < 0.05) and |log_2_FC| > 2. Results of differential abundance were visualized using the EnhancedVolcano package. Correlation and visualization of the obtained results were conducted using Python. Data processing was performed with Numpy and Pandas libraries Matplotlib and Seaborn libraries were utilized for graph plotting. Spearman's correlation coefficients were calculated using the scipy.stats library with P-values adjusted using the Benjamini-Hochberg procedure *via* the statsmodels package.

## 3 Results

### 3.1 Clinical characteristics of the study groups

A study was conducted to investigate the differences in main clinical and laboratory parameters between groups. Significant differences were identified between the groups with regard to the presence of hypertension. In the Control group, the prevalence was 67%, while in the Obesity-IHD group it was 99% (p=0.001). Furthermore, the prevalence of ACS was higher in the Obesity-IHD group than in the control group (0% vs. 49%, p=0.001). However, there was no significant difference in the prevalence of stroke or smoking status among the study groups (p=0.2). The data are presented in **Table 1** and **Supplementary Table S1**.

**Table 1:** The baseline characteristics of the study groups (categorical parameters).

Parameter	Control n=27	Obesity n=59	IHD n=22	Obesity- IHD n=74	p value Overall
Sex, N (%)	M — 5 (19%) F — 22 (81%)	M — 7 (12%) F — 52 (88%)	M — 5 (23%) F — 22 (77%)	M — 25 (34%) F — 49 (66%)	0.003
Hypertensio n, N (%)	18 (67%)	56 (95%)	15 (68%)	73 (99%)	0.001
Acute coronary syndromes, N (%)	0 (0%)	0 (0%)	8 (36%)	36 (49%)	0.001
Stroke, N (%)	0 (0%)	0 (0%)	2 (9%)	9 (12%)	0.08
Impaired glucose tolerance N (%)	1 (4%)	10 (17%)	1 (5%)	27 (36%)	0.001
Smoking, N (%)	4 (15%)	9 (15%)	0 (0%)	8 (11%)	0.2
Use of statins N (%)	8 (30%)	16 (27%)	13 (59%)	56 (76%)	0.001

The parameters of blood pressure were found to be significantly correlated with the presence of obesity. Both systolic and diastolic blood pressure were elevated in the Obesity (p=0.027 and 0.044, respectively) and Obesity-IHD (p=0.005 and 0.046, respectively) groups compared to the Control group. Biochemical parameters revealed notable differences in glucose levels, which were higher in the Obesity group than in the IHD group (p=0.010). Additionally, total cholesterol (TC) levels were lower in the Obesity-IHD group compared to the Control, Obesity, and IHD groups (p=0.001, 0.001 and 0.015 respectively), which can be attributed to the more prevalent use of statins in this group. Comparable trends were observed for low-density lipoproteins (LDL), high-density lipoproteins (HDL), and non-HDL parameters. Carotid intima-media thickness (cIMT) was significantly elevated in the IHD and Obesity-IHD groups. The data are presented in **Table 2** and **Supplementary Table S2**.

**Table 2:** The baseline characteristics of study groups (continuous parameters). Data are presented as median and interquartile range.

Parameter	Control n=27	Obesity n=59	IHD n=22	Obesity- IHD n=74	p value Overall
Age	58.0 (54.0, 60.0)	57.0 (54.0, 62.0)	67.5 (62.0,71.0)	63.0 (58.0, 68.0)	0.001
Height, cm	164 (161, 170)	163 (158, 168)	164 (160, 170)	165 (158, 174)	0.5
Body weight, kg	71 (66, 82)	95 (88, 104)	74 (70, 80)	108 (92, 117)	0.001
BMI, kg/m^2^	27 (26, 29)	36 (33, 41)	28 (24, 30)	38 (35, 44)	0.001
Fat mass, kg	26 (24, 32)	45 (39, 54)	28 (21, 34)	50 (39, 60)	0.001
VAT area, cm^2^	141 (125, 171)	231 (210, 249)	142 (97, 187)	232 (206, 265)	0.001
sBP, mmHg	120 (110, 130)	130 (120, 130)	120 (110, 130)	130 (120, 138)	0.013
dBP, mmHg	80 (70, 80)	80 (80, 90)	80 (80, 80)	80 (80, 90)	0.2
Glucose, mmol/L	5.05 (4.65, 5.22)	5.20 (4.81, 5.51)	4.79 (4.55,4.97)	5.48 (4.90, 5.90)	0.001
TC, mmol/L	5.72 (5.25, 6.30)	5.54 (4.70, 6.26)	5.55 (4.47,6.49)	4.37 (3.66, 5.29)	0.001
TG, mmol/L	1.15 (0.79, 1.48)	1.29 (1.01, 1.65)	0.84 (0.68, 1.51)	1.32 (0.89, 1.71)	0.091
LDL cholesterol, mmol/L	3.59 (2.88,4.38)	3.75 (2.81, 4.30)	3.82 (2.77, 4.56)	2.73 (2.31, 3.49)	0.001
HDL cholesterol, mmol/L	1.56 (1.31, 1.91)	1.42 (1.14, 1.60)	1.54 (1.10, 1.72)	1.22 (0.98, 1.46)	0.001
Non-HDL mmol/L	3.95 (3.31, 4.72)	4.31 (3.41, 4.71)	3.92 (3.20, 5.08)	3.11 (2.40, 4.13)	0.001
cIMT, mm	0.73 (0.66, 0.83)	0.73 (0.67, 0.83)	0.80 (0.74, 0.86)	0.86 (0.78, 0.94)	0.001

BMI, body mass index; dBP, diastolic blood pressure; sBP, systolic blood pressure; cIMT, carotid intima-media thickness; HDL, high-density lipoproteins; LDL, low-density lipoproteins; TC, total cholesterol; TG, triglycerides; VAT, visceral adipose tissue.

### 3.2 Comparative analysis of the structure of the intestinal and oral microbiota

The composition of intestinal and oral microbiota was analyzed using 16S rRNA sequencing. In the gut microbiome, the dominant phyla were *Firmicutes (Bacillota), Bacteroidetes (Bacteroidota)*, and *Proteobacteria (Pseudomonadota).* The *Firmicutes* demonstrated the highest relative abundance, averaging 69.5% across all groups, while the *Proteobacteria* represented 16.8% and the *Bacteroidetes* contributed 11.3%. It is noteworthy that there was a trend toward a relative decrease in *Proteobacteria* in obese and ischemic obese individuals compared to control group. Additionally, the phyla *Verrucomicrobia* (*Verrucomicrobiota)* and *Actinobacteria (Actinomycetota)* accounted for 1.2% and 0.6%, respectively.

The oral microbiome exhibited distinctive patterns, with prevailing phyla *Firmicutes*, *Proteobacteria*, and *Bacteroidetes*. *Firmicutes* remained the most abundant phylum, with an average relative abundance of 57.8%. In contrast, *Proteobacteria* and *Bacteroidetes* accounted for approximately 21.8% and 12.6%, respectively. Notably, increased *Proteobacteria* were observed in saliva samples from individuals with IHD and obesity compared to control, indicating potential changes in oral bacterial composition associated with these conditions. Additionally, *Fusobacteria* were also present at lower levels.

**Figure 2:**
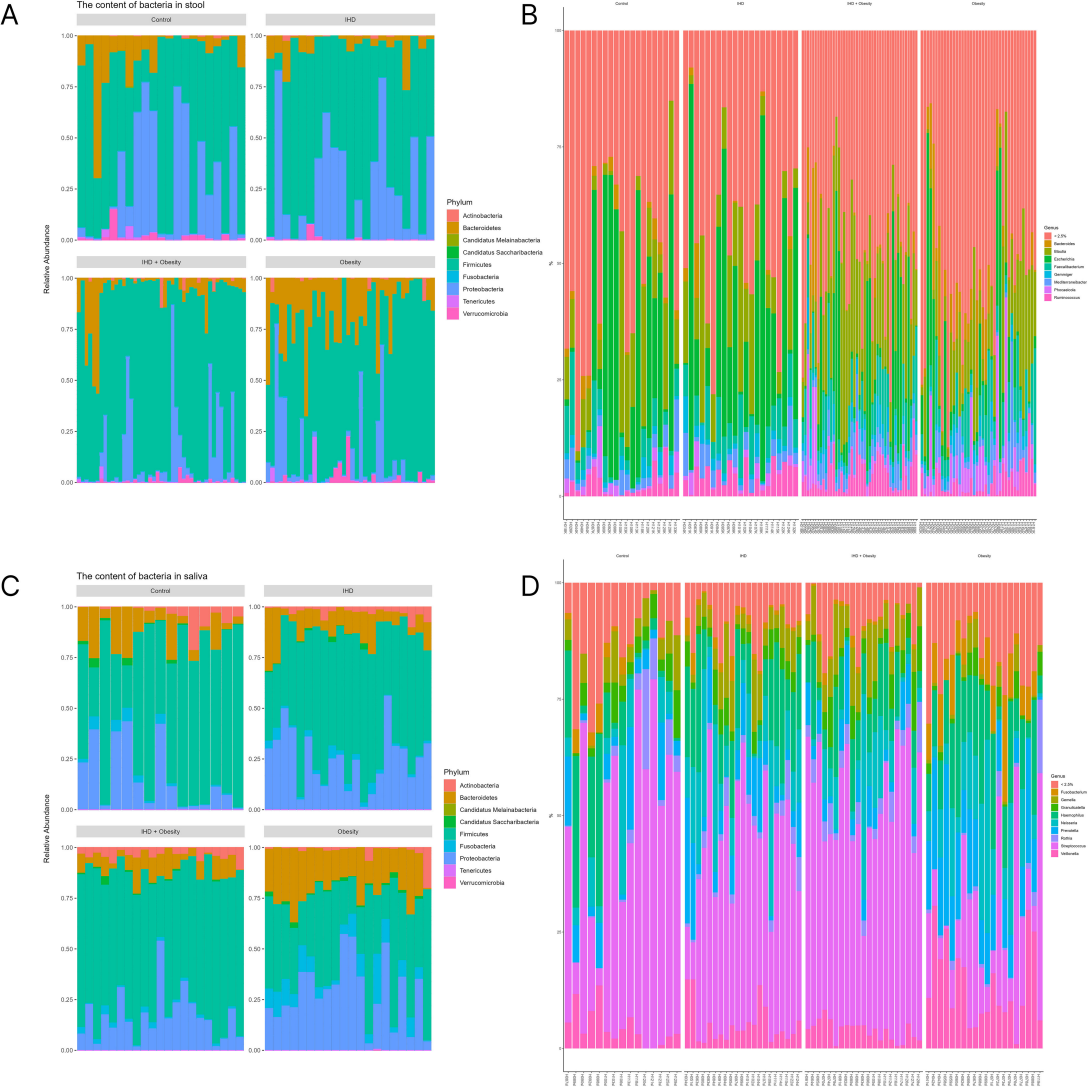
Gut and oral microbiota composition. The most represented phyla and genera of the gut microbiome are provided in **(A, B)**, while the most represented phyla and genera of the oral microbiome are provided in **(C, D)**. The data are presented as relative abundance for each sample.

At the genus level, notable differences were observed between the groups. The gut microbiome was dominated by the genera *Bacteroides*, *Blautia*, *Escherichia*, *Faecalibacterium*, and *Ruminococcus*. In contrast, the oral microbiome structure was dominated by the genera *Streptococcus*, *Prevotella*, *Fusobacteria*, and *Veillonella*. The results are presented in **Figure 2**.

**Figure 3:**
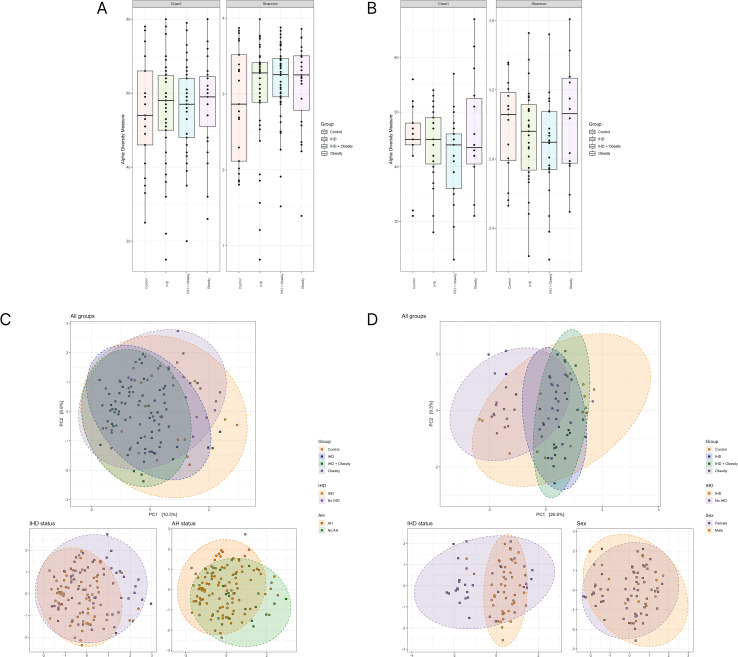
Comparative analysis of alpha and beta diversity of the intestinal **(A, C)** and oral **(B, D)** microbiota between study groups. Alpha diversity was assessed using metrics: Chao1 index, Shannon index. Beta diversity of bacteria was identified with the principal coordinates analysis (PCoA).

A comparative analysis was conducted to ascertain whether variations existed in the structure of the gut and oral microbiota among the study groups. Alpha diversity was evaluated using the Chao1 and Shannon indices, and no notable differences were observed in either the oral or gut microbiomes across the groups. To investigate beta diversity, a principal component analysis was conducted. Significant differences in beta diversity were observed between Control and Obesity-IHD groups (p=0.006), IHD and Obesity groups (p=0.008), and Obesity and Obesity-IHD groups (p=0.006) in the gut microbiome. Similar data were identified for the oral microbiota, with statistically significant differences observed between Control and IHD groups (p = 0.012), Control and Obesity groups (p = 0.003), and Obesity and Obesity-IHD groups (p = 0.048). Furthermore, significant differences were observed between IHD and Obesity groups (p = 0.004) and between Obesity and Obesity-IHD groups (p = 0.003). These findings suggest that while alpha diversity remained consistent across groups, beta diversity of gut and oral microbiota differed significantly among certain groups, reflecting potential links between microbial community composition, obesity, and IHD. The results are presented in **Figure 3**.

**Figure 4:**
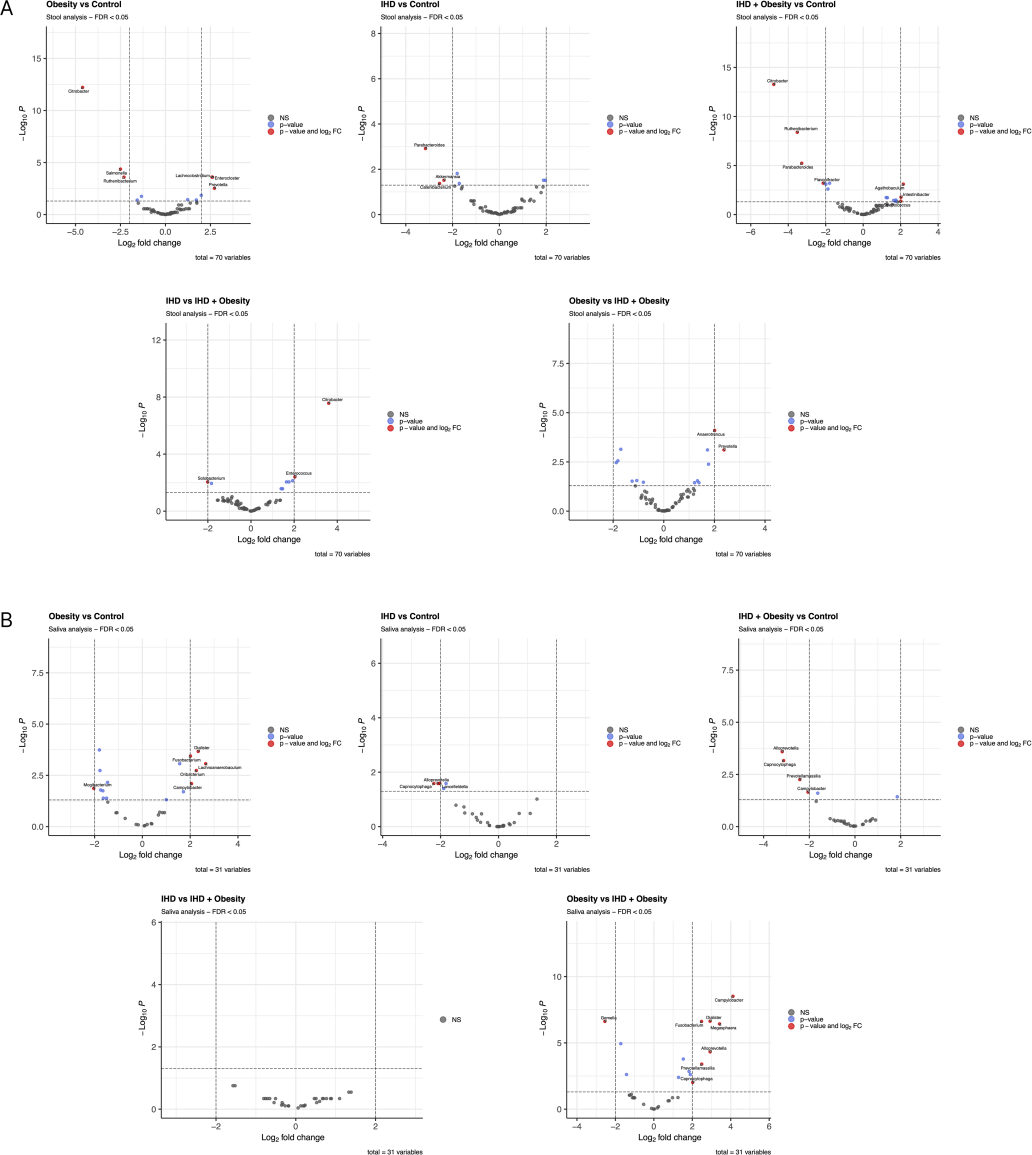
DESeq2 differential abundance analysis of genera in study groups of intestinal **(A)** and oral **(B)** microbiota. Data are presented on a volcano plot with thresholds of padj < 0.05 and |log_2_FC| > 2.

Distinct patterns in the relative abundance of microbial genera were observed among the study groups (**Figure 4A**). In the gut microbiome of the Obesity group, the abundances of *Lachnoclostridium*, *Enterocloster*, and *Prevotella* were higher, whereas the levels of *Citrobacter*, *Salmonella*, and *Ruthenibacterium* were lower compared to the Control group. In the IHD group, the abundances of *Parabacteroides*, *Catenibacterium*, and *Akkermansia* were reduced relative to the Control group. In the Obesity-IHD group, the abundances of *Streptococcus*, *Intestinibacter*, and *Agathobaculum* were increased, while *Citrobacter*, *Ruthenibacterium*, *Parabacteroides*, and *Flavonifractor* were decreased compared to the Control group. A comparison between the IHD and Obesity-IHD groups revealed that *Enterococcus* and *Citrobacter* were more abundant in the IHD group, whereas *Solobacterium* was less abundant. Furthermore, relative to the Obesity group, the Obesity-IHD group showed higher levels of *Anaerotruncus* and *Prevotella*.

In the oral microbiome, variations in the relative abundance of bacterial genera were apparent among the study groups. The Obesity group showed higher levels of *Fusobacterium*, *Campylobacter*, *Oribacterium*, *Dialister*, and *Lachnoanaerobaculum*, while *Mogibacterium* was decreased relative to the Control group. The IHD group exhibited reduced levels of *Capnocytophaga*, *Alloprevotella*, and *Lancefieldella* compared to the Control group. In the Obesity-IHD group, a reduction in *Alloprevotella*, *Capnocytophaga*, *Prevotellamassilia*, and *Campylobacter* was noted in comparison with the Control group. A comparison between the Obesity-IHD and IHD groups revealed no significant differences in the relative abundance of bacterial genera. However, when compared to the Obesity group, the Obesity-IHD group demonstrated decreased levels of *Gemella* and increased levels of *Capnocytophaga*, *Fusobacterium*, *Prevotellamassilia*, *Dialister*, *Alloprevotella*, *Megasphaera*, and *Campylobacter* (**Figure 4B**).

### 3.3 Analysis of associations between gut and oral microbiome composition and blood biochemical parameters, body composition parameters

**Figure 5:**
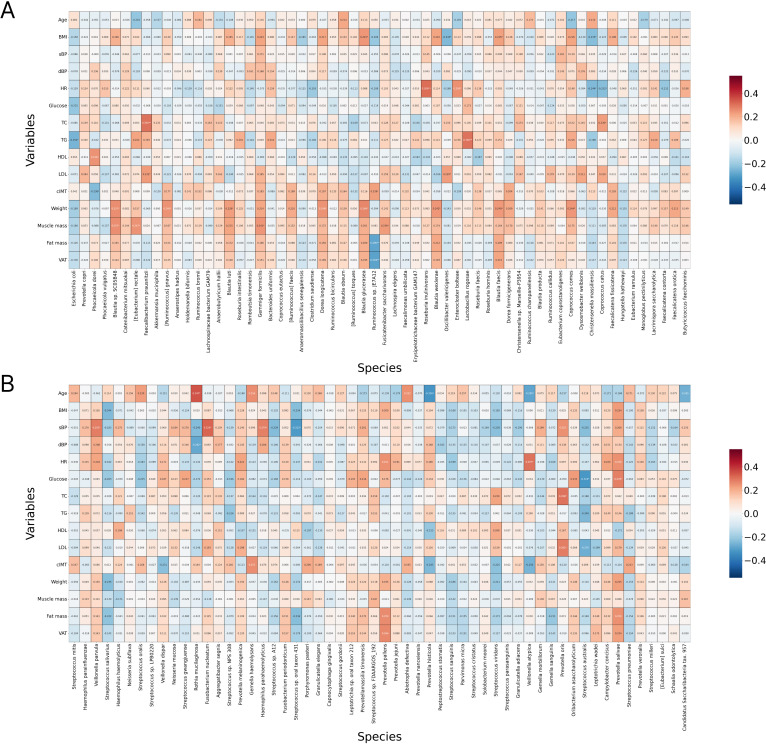
Spearman correlation between the relative abundance of gut **(A)** and oral **(B)** bacteria and body composition, serum glucose and lipids. P-values were adjusted for multiple testing using the Benjamini-Hochberg procedure; *p < 0.05, **p < 0.01.

Spearman correlation analysis was performed to investigate potential associations between microbial abundance and various clinical parameters. A positive correlation was observed between the gut bacteria *Blautia glucerasea* and *Blautia faecis* with body mass index (BMI), while a negative correlation was noted between *Oscillibacter valericigenes* and BMI. *Ruminococcus* sp was linked to lower visceral adipose tissue (VAT) area. Higher levels of *Faecalibacterium prausnitzii* and *Coprococcuscatus* were associated with increased TC. Additionally, *Lactobacillus rogosae* was found to correlate with elevated TG levels, whereas *Escherichia coli* was negatively associated with TG concentrations*. Faecalibacterium prausnitzii* and *Oscillibacter valericigenes* were associated with increased LDL cholesterol levels. In addition, elevated levels of *Phocaeicola dorei* were found to correlate with higher levels of HDL (**Figure 5A**).

The prevalence of certain oral microbiota representatives was also found to correlate with various anthropometric and biochemical parameters. Specifically, *Veillonella parvula*, *Fusobacterium nucleatum*, and *Haemophilus parahaemolyticus* were associated with higher blood pressure, while *Streptococcus* sp. and *Rothia mucilaginosa* were associated with lower blood pressure. Additionally, *Prevotella oris* was connected to elevated levels of TC and LDL cholesterol. Higher prevalence of *Streptococcus australis* was associated with decreased glucose levels, while *Prevotella salivae* was associated with an increase one (**Figure 5B**).

## 4 Discussion

The present study revealed differences in the composition of the gut and oral microbiome between the groups of participants with obesity and IHD. However, no significant differences in alpha diversity were observed between the groups in either the gut or oral microbiomes. According to the meta-analysis, only 9 of the 22 studies reported differences in alpha diversity between obese and normal-weight adults (Pinart et al., 2021b). The effects of obesity on oral microbiome alpha diversity remain controversial, with one study indicating that obesity is associated with a significant reduction in alpha diversity and another study finding no difference (Yang et al., 2019).

At the same time, considerable discrepancies in beta diversity were identified between Control and Obesity-IHD groups, IHD and Obesity groups, and Obesity and Obesity-IHD groups within the gut microbiome. Similar data were identified for the oral microbiota, with statistically significant differences observed between Control and IHD groups, Control and Obesity groups, and Obesity and Obesity-IHD groups. Moreover, notable discrepancies were identified between IHD and Obesity groups, as well as between Obesity and Obesity-IHD groups. In a study examining the structure of intestinal and oral microbiomes, significant differences in beta diversity were revealed for both microbiomes (Stefura et al., 2021). Furthermore, there is evidence to suggest that obesity influences beta diversity in women with periodontitis (Thomas et al., 2021).

Additionally, significant intergroup differences were observed in the composition of microbiota genera. Individuals with obesity had higher levels of *Lachnoclostridium*, *Enterocloster*, and *Prevotella*, and lower levels of *Citrobacter*, *Salmonella*, and *Ruthenibacterium*. IHD was associated with lower levels of *Parabacteroides*, *Catenibacterium*, and *Akkermansia*. Among participants with both obesity and IHD, higher abundances of *Streptococcus*, *Intestinibacter*, and *Agathobaculum*, as well as lower levels of *Citrobacter*, *Ruthenibacterium*, *Parabacteroides*, and *Flavonifractor*, were observed. A recent study using Mendelian randomization showed that *Lachnoclostridium* abundance is associated with an increased risk of T2D, presumably through its role in blood glucose regulation and abdominal fat accumulation (Fu et al., 2025). Another study showed that increased *Lachnoclostridium* in the gut was, in contrast, associated with a reduced risk of NAFLD (Dai et al., 2023). In addition, it has been demonstrated that *Lachnoclostridium* may promote atherosclerosis through TMAO production (Cai et al., 2022). *Enterocloster* abundance in a recent study was reduced after 3 weeks of intermittent fasting and weight loss (Hu et al., 2023). The *Prevotella* genus, as well as a high *Prevotella*/*Bacteroides* ratio, have been associated with increased BMI in the literature (Xu et al., 2022). Notably, a recent study demonstrated a correlation between a higher *Prevotella/Bacteroides* ratio and accelerated weight loss when a high-fiber diet was employed (Hjorth et al., 2019). A study in obese children showed that *Citrobacter* was negatively correlated with pro-inflammatory cytokines, particularly TNF-a (Yuan et al., 2021). A study examining the effects of a complete 10-day fast in adult men showed that the abundance of *Ruthenibacterium lactatiformans* increased significantly following such restrictions and corresponding weight loss (Wu et al., 2024). Furthermore, there is evidence indicating an elevated risk of IHD in individuals with hypertension and an increased abundance of *Prevotella* and *Klebsiella* in the intestine (Li et al., 2017). The study also revealed that the prevalence of *Streptococcus* and *Klebsiella* was significantly higher in patients with symptomatic carotid atherosclerosis compared to healthy individuals (Lv et al., 2024). A systematic review of 16 studies found several patterns in the gut microbiome composition in obese individuals, including increased abundance of the genus *Streptococcus* (Díaz Perdigones et al., 2025). The concentration of *Solobacterium* was found to be higher in the group of individuals with obesity and diabetes compared to those with obesity alone. Furthermore, a positive correlation was observed between the concentration of *Solobacterium* and the HbA1c concentration (Shinoda et al., 2024). Moreover, an *in vivo* study demonstrated that gut *Parabacteroides merdae* may exert a protective effect on cardiovascular disease by enhancing the catabolism of branched-chain amino acids (BCAAs) (Qiao et al., 2022). Additionally, *Catenibacterium* were shown to be depleted in patients with a high cardiovascular risk score (Kelly et al., 2016). For patients with coronary artery disease, depletion of *Akkermansia muciniphila* was shown to correlate with an unsatisfactory response to statin therapy (Wang et al., 2021).

The oral microbiome exhibited notable differences in the relative abundance of bacterial genera between the study groups. The prevalence of Fusobacterium, Campylobacter, *Oribacterium*, *Dialister*, and *Lachnoanaerobaculum* was higher in obese individuals, while *Mogibacterium* was less prevalent. The levels of *Capnocytophaga*, *Alloprevotella*, and *Lancefieldella* were found to be reduced in patients with IHD. A reduction in the prevalence of *Alloprevotella*, *Capnocytophaga*, *Prevotellamassilia*, and *Campylobacter* was observed in individuals with obesity and IHD. According to the literature, *Fusobacterium* and, in particular, *Fusobacterium nucleatum* are more strongly associated with an increased risk of developing cancer (Fan et al., 2023). However, with regard to the relationship between this bacterium and obesity, the available data are inconclusive (Narii et al., 2022). The study demonstrated that *Porphyromonas gingivalis* and *F. nucleatum* were more prevalent in individuals with a healthy BMI (Rahman et al., 2023). Furthermore, *F. nucleatum* has been demonstrated to accelerate aortic inflammation and atherosclerosis in the aorta of ApoE-null mice (Velsko et al., 2015). The study found that *Oribacterium* was more prevalent among individuals with a BMI above 40 (Stefura et al., 2021). Additionally, another study has shown that *Oribacterium* may affect the sense of taste (Cattaneo et al., 2019). It is noteworthy that a recent study observed a reduction in the genus *Alloprevotella* among participants with a BMI greater than 50, in comparison to other individuals with obesity (Stefura et al., 2021). A study of 1,049 residents of Atlantic Canada found that *Mogibacterium* was associated with higher waist size, while *Prevotella* was associated with higher waist size and weight (Nearing et al., 2020). Increased abundance of *Dialiste*r invisus has been shown to be associated with obesity among periodontally healthy individuals (Khocht et al., 2023).

Correlation analysis revealed that within the gut microbiome, representatives of the genus *Blautia* were associated with higher BMI, while *Oscillibacter valericigenes* exhibited a negative correlation with BMI. The presence of *Ruminococcus* sp. was found to be associated with a reduction in VAT. *Faecalibacterium prausnitzii* was found to be associated with dyslipidemia. At present, there is a tendency to consider *Blautia* as a beneficial bacterium with a favorable influence on obesity and metabolic syndrome (Chanda et al., 2024). Nevertheless, the data are inconclusive. Consequently, the abundance of *Blautia* was demonstrated to decline, while that of *Bacteroides* increased following six months of bariatric surgery (Kim et al., 2022). In a separate study, a negative correlation was observed between *Blautia* abundance and VAT, irrespective of gender (Ozato et al., 2019). An association was observed between elevated *Oscillobacter* levels and reduced blood triglyceride concentrations (Liu et al., 2022).

Among the oral microbiome representatives, *Veillonella parvula*, *Fusobacterium nucleatum*, *Haemophilus parahaemolyticus*, and *Prevotella oris* were associated with elevated blood pressure, whereas the presence of *Streptococcus* sp. and *Rothia mucilaginosa* was associated with reduced blood pressure. *Prevotella oris* was connected with elevated levels of TC and LDL cholesterol. *F. nucleatum* is thought to be primarily associated with cancer, but some studies have shown that it may play a role in vascular diseases such as cerebral small vessel disease and atherosclerosis (Luo et al., 2024a; Aoki et al., 2025). A study in older women found that depletion of *Prevotella* and *Streptococcus oralis* was associated with increased blood pressure (Gordon et al., 2019).

Intestinal microbiota may influence obesity by increasing energy harvesting and signaling *via* short-chain fatty acids through GPR41/43, which regulates adipose storage and gut hormones. They also transform bile acids, which engage FXR/TGR5 and enhance GLP-1 secretion (Overby and Ferguson, 2021; Masse and Lu, 2023). Dysbiosis has been demonstrated to result in impaired barrier integrity and elevated circulating LPS. Elevated LPS has been shown to provoke metabolic endotoxemia, which in turn has been demonstrated to drive low-grade inflammation, insulin resistance, and adiposity (Breton et al., 2022). The oral microbiota also contributes to the oral–gut axis, where *P. gingivalis* can translocate to the intestine, disrupt tight junctions, and shift gut taxa. This can impair skeletal muscle glucose uptake and exacerbate diet-induced obesity and insulin resistance in mice (Niu et al., 2024). Additional oral mechanisms include systemic low-grade inflammation, and effects on taste and appetite. These mechanisms link oral dysbiosis to adipose dysfunction and broader metabolic disease (Schamarek et al., 2023).

The search for new biomarkers for diagnostics and therapeutic targets is a pressing area of ​​modern research. A comprehensive assessment of microbiomes, metabolites, microRNAs, and other molecular parameters allows for a deeper understanding of the pathogenesis of various diseases. This integrative approach helps to identify relationships between microbiota status, metabolic changes, and gene regulation, opening up new opportunities for the development of personalized diagnostic and treatment strategies (Superko et al., 2011; Tocia et al., 2023).

This study has several limitations, including unequal sample sizes in group comparisons. There is also an age disparity, but no differences in age categories. Additionally, this study did not control for several potential influencing factors, such as diet, medications not listed in the exclusion criteria, and comorbidities. The groups differed in statin use, which is explained by the necessity of their use in individuals with IHD and high cardiovascular risk. Statin use may potentially influence the composition of the microbiome, but no definitive patterns have been established (Dias et al., 2020; Dharmarathne et al., 2024). The cross-sectional design of the study does not allow for conclusions to be drawn about cause-and-effect relationships.

## 5 Conclusions

In conclusion, our study provides evidence that obesity and IHD are associated with distinct alterations in both gut and oral microbiota compositions. Future research should focus on elucidating the mechanistic pathways through which specific microbial taxa influence metabolic health, potentially leading to the development of microbiota-targeted therapies for obesity and cardiovascular diseases.

## Data Availability

The datasets presented in this study can be found in online repositories. The names of the repository/repositories and accession number(s) can be found below: https://www.ncbi.nlm.nih.gov/, PRJNA1306337.
