# Supplementary material for: Gut and oral microbiome profiles in patients with obesity and ischemic heart disease
Source: Front Cell Infect Microbiol. 2025 Dec 12;15:1695279. doi: 10.3389/fcimb.2025.1695279 (PMC12740868; doi:10.3389/fcimb.2025.1695279)
Supplement: Supplementary file 1 [file Table1.pdf]

Table S1. The baseline characteristics of the study groups (categorical parameters), including pairwise comparisons.

**Parameter**	**Control** n=27 (1)	**Obesity** n=59 (2)	**IHD** n=22 (3)	**Obesity-IHD** n=74 (4)	**p value 1-2**	**p value 1-3**	**p value 2-3**	**p value 1-4**	**p value 2-4**	**p value 3-4**	**p value Overall**
Sex, N (%)	M — 5 (19%) F — 22 (81%)	M — 7 (12%) F — 52 (88%)	M — 5 (23%) F — 22 (77%)	M — 25 (34%) F — 49 (66%)	0.411	0.719	0.224	0.055	0.05	0.162	0.003
Hypertension, N (%)	18 (67%)	56 (95%)	15 (68%)	73 (99%)	0.001	0.911	0.001	0.001	0.212	0.001	0.001
Acute coronary syndromes, N (%)	0 (0%)	0 (0%)	8 (36%)	36 (49%)	0.965	0.001	0.001	0.001	0.001	0.319	0.001
Stroke, N (%)	0 (0%)	0 (0%)	2 (9%)	9 (12%)	0.965	0.113	0.726	0.059	0.043	0.693	0.08
Impaired glucose tolerance N (%)	1 (4%)	10 (17%)	1 (5%)	27 (36%)	0.090	0.883	0.150	0.001	0.013	0.004	0.001
Smoking, N (%)	4 (15%)	9 (15%)	0 (0%)	8 (11%)	0.958	0.062	0.053	0.586	0.447	0.109	0.2
Use of statins N (%)	8 (30%)	16 (27%)	13 (59%)	56 (76%)	0.811	0.040	0.008	0.001	0.001	0.131	0.001

Table S2. The baseline characteristics of study groups (continuous parameters), including pairwise comparisons. Data are presented as median and interquartile range.

Parameter	Control n=27 (1)	Obesity n=59 (2)	IHD n=22 (3)	Obesity-IHD n=74 (4)	p value 1-2	p value 1-3	p value 2-3	p value 1-4	p value 2-4	p value 3-4	p value Overall
Age	58.0 (54.0, 60.0)	57.0 (54.0, 62.0)	67.5 (62.0, 71.0)	63.0 (58.0, 68.0)	0.9	0.001	0.001	0.020	0.001	0.077	0.001
Height, cm	164 (161, 170)	163 (158, 168)	164 (160, 170)	165 (158, 174)	0.9	0.9	0.9	0.9	0.9	0.9	0.5
Body weight, kg	71 (66, 82)	95 (88, 104)	74 (70, 80)	108 (92, 117)	0.001	0.9	0.001	0.001	0.021	0.001	0.001
BMI, kg/m^2	27 (26, 29)	36 (33, 41)	28 (24, 30)	38 (35, 44)	0.001	0.9	0.001	0.001	0.4	0.001	0.001
Fat mass, kg	26 (24, 32)	45 (39, 54)	28 (21, 34)	50 (39, 60)	0.001	0.9	0.001	0.001	0.9	0.001	0.001
VAT area, cm^2	141 (125, 171)	231 (210, 249)	142 (97, 187)	232 (206, 265)	0.001	0.9	0.001	0.001	0.9	0.001	0.001
sBP, mmHg	120 (110, 130)	130 (120, 130)	120 (110, 130)	130 (120, 138)	0.027	0.9	0.7	0.005	0.9	0.7	0.013
dBP, mmHg	80 (70, 80)	80 (80, 90)	80 (80, 80)	80 (80, 90)	0.044	0.9	0.9	0.046	0.9	0.9	0.2
Glucose, mmol/L	5.05 (4.65, 5.22)	5.20 (4.81, 5.51)	4.79 (4.55, 4.97)	5.48 (4.90, 5.90)	0.137	0.06	0.010	0.056	0.4	0.001	0.001
TC, mmol/L	5.72 (5.25, 6.30)	5.54 (4.70, 6.26)	5.55 (4.47, 6.49)	4.37 (3.66, 5.29)	0.9	0.9	0.9	0.001	0.001	0.015	0.001

TG, mmol/L	1.15 (0.79, 1.48)	1.29 (1.01, 1.65)	0.84 (0.68, 1.51)	1.32 (0.89, 1.71)	0.6	0.9	0.2	0.9	0.9	0.4	0.091
LDL cholesterol, mmol/L	3.59 (2.88, 4.38)	3.75 (2.81, 4.30)	3.82 (2.77, 4.56)	2.73 (2.31, 3.49)	0.9	0.9	0.9	0.12	0.003	0.029	0.001
HDL cholesterol, mmol/L	1.56 (1.31, 1.91)	1.42 (1.14, 1.60)	1.54 (1.10, 1.72)	1.22 (0.98, 1.46)	0.045	0.9	0.9	0.001	0.2	0.043	0.001
Non-HDL mmol/L	3.95 (3.31, 4.72)	4.31 (3.41, 4.71)	3.92 (3.20, 5.08)	3.11 (2.40, 4.13)	0.9	0.9	0.9	0.031	0.001	0.079	0.001
cIMT, mm	0.73 (0.66, 0.83)	0.73 (0.67, 0.83)	0.80 (0.74, 0.86)	0.86 (0.78, 0.94)	0.9	0.9	0.6	0.005	0.001	0.12	0.001

Abbreviations: BMI = body mass index; dBP = diastolic blood pressure; sBP = systolic blood pressure; cIMT = carotid intima-media thickness; HDL = high-density lipoproteins; LDL = low-density lipoproteins; TC = total cholesterol; TG = triglycerides; VAT = visceral adipose tissue.
